# A Review of Selected Studies That Determine the Physical and Chemical Properties of Saliva in the Field of Dental Treatment

**DOI:** 10.1155/2018/6572381

**Published:** 2018-05-09

**Authors:** Elżbieta Kubala, Paulina Strzelecka, Marta Grzegocka, Danuta Lietz-Kijak, Helena Gronwald, Piotr Skomro, Edward Kijak

**Affiliations:** ^1^Doctorate Studies at Pomeranian Medical University in Szczecin, Rybacka 1, 70-204 Szczecin, Poland; ^2^Independent Laboratory of Propaedeutic and Dental Physical Diagnostics, Pomeranian Medical University in Szczecin, Rybacka 1, 70-204 Szczecin, Poland; ^3^Scientific Laboratory of Dysfunction of the Masticatory System, Chair and Department of Prosthodontics, Pomeranian Medical University in Szczecin, Rybacka 1, 70-204 Szczecin, Poland

## Abstract

Physiological whole saliva is a unique body fluid constantly washing the mucous membranes of the mouth, throat, and larynx. Saliva is a clear, slightly acidic mucinous-serous secretion, composed of various electrolytes, small organic substances, proteins, peptides, and polynucleotides. There are many ways to use saliva as a biological fluid (biofluid). The significant advantages of saliva as a unique diagnostic material are its availability and the noninvasive method of collection. The aim of this review is to emphasize the diagnostic value of saliva as a research material in the configuration of its structure and secretion disorders. The data were obtained using the MEDLINE (PubMed) search engine, as well as an additional manual search. The analysis covered 77 articles selected from a group of 1986 publications and initially qualified for devising. The results were evaluated and checked for the correctness of qualifying in accordance with inclusion and exclusion criteria. The diagnostic use of saliva has attracted the attention of many researchers due to its noninvasive nature and relative simplicity of collection. In addition, it should be noted that the determination of chemical and physical saliva parameters can be effectively performed in the patient's presence in the dental office.

## 1. Introduction

Physiological whole saliva (WS) is a unique body fluid constantly washing the mucous membranes of the mouth, throat, and larynx. Apart from the gums and the anterior part of the hard palate, the entire oral mucosa contains fine salivary glands (from 200 to 400) which are responsible for the production of only 10% of the secretion. The large glands include 3 pairs of salivary glands, parotid, submandibular, and sublingual, which produce 90% of saliva [1]. These glands can also be classified in terms of the type of secretion produced as serous, mucus, and mixed. Saliva is a clear, slightly acidic mucinous-serous secretion, composed of various electrolytes, small organic substances, proteins, peptides, and polynucleotides [2]. About 65% of unstimulated (resting) saliva comes from the submandibular gland, 25% from the parotid gland, 4% from the sublingual gland, and 8% from other salivary glands [3].

Given the integrity of the hard and soft tissues of the oral cavity, this secretion is extremely important and constitutes a factor which is the most relevant to the maintenance of homeostasis in the mouth (due to the content of organic and inorganic components). It moisturizes oral tissues, allowing for articulation, digestion, and swallowing [4]. The fluid is also responsible for the protection of the surface of the teeth and mucous membranes against biological, mechanical, and chemical factors [5]. It participates in the perception of taste stimuli, temperature, and touch. The protective function of saliva is manifested in the removal of harmful products of bacterial metabolism, bacteria, and food debris from the oral cavity and the surface of the teeth. The purification rate may vary from 0.8 to 8 ml/min. It is lower for the surfaces that are difficult for saliva to access. Saliva moistens the mucous membranes and teeth. Its proteins cover them with a thin coat, called the pellicle, which consists of amino acids and proteins. These substances are selectively absorbed on the surface of the teeth as a result of interaction with the enamel hydroxyapatite [6]. Moistening facilitates the formation and swallowing of bites and chewing of food, as well as reducing the harmful effects of mechanical, chemical, thermal, and biological injuries on the mucous membranes [4].

Saliva contains 99.5% water, 0.3% protein, and 0.2% inorganic and organic substances [7]. The most common inorganic constituents are sodium, potassium, calcium, magnesium, chlorides, and carbonates, while organic components include amylases, peroxidases, lipases, mucins, lysozyme, lactoferrin, kallikreins, cystatins, hormones, and growth factors [8]. In a healthy individual, daily salivation is estimated at 0.5 to 2 litres [9]. The regulation of salivary secretion takes place through the nervous pathway via the cholinergic system and a and b fibres of the sympathetic nervous system. While asleep, the rate of salivary secretion decreases and during chewing or speech it increases substantially. The secretion rate varies widely from person to person, even under normal conditions. After strong excitatory stimulation, for example, while eating, the salivation rate can increase markedly and after the administration of pharmacological agents the value can double [10].

Mucins (MUC) present in saliva protect the surface of oral mucous membranes from toxins and various types of irritants contained in stimulants or food. Recent studies also prove that these substances protect the mouth by various mechanisms influenced by unique polymer structures. First, mucins can interact with salivary proteins to change their location and retention and thus increase the protection of the mouth. In addition, MUC7 and MUC5B may interact with microorganisms in the oral cavity to facilitate their removal and/or reduce their pathogenicity [11]. However, such factors as neutral pH and the presence of Ca2+ and Mg2+ ions facilitate the healing of abrasions and wounds of the mucous membrane.

Saliva also plays a very important role in the inhibition and development of carious lesions of the teeth, improving remineralization of the tooth enamel and preventing demineralization [5]. When the pH of this secretion is within 6.8–7.2, it becomes a saturated solution of calcium phosphates, which results in quick and effective remineralization of the initial changes. However, if we slightly acidify the environment, saliva becomes an unsaturated solution and easily soluble calcium hydrogen phosphates are formed; thus the susceptibility of the teeth to caries increases [6]. Specific defensive factors include immunoglobulins: IgA (affecting phagocytosis of streptococci by leukocytes), IgG (together with IgA slow down the formation of tartar), and IgM (partially produced by the parotid, their presence indicates the existence of acute inflammation). In the group of nonspecific defensive factors, we can distinguish enzymes and bactericidal substances, such as lysozyme, lactoferrin, histatins, mucins, and salivary peroxidase [8]. Saliva also contains buffer systems responsible for maintaining proper acid-base balance. The most important role is played by the bicarbonate buffer. The buffers maintain the pH of resting saliva between 5.7 and 6.2, while the pH of stimulated saliva can reach 8 [6].

Inorganic ingredients of saliva come mainly from blood. Their content in the secretion is not constant and they always appear in the ionized form. Cations such as Na+ and K+ are involved in the active transport of compounds through cell membranes, while Ca2+ and Mg2+ activate some enzymes. The Cl^−^ anion activates *α*-amylase and F^−^ has anticarious activity, whereas I^−^ plays a role in defence mechanisms, mainly due to the presence of peroxidase in the system [12].

There are many ways to use saliva as a biological fluid (biofluid). The significant advantages of saliva as a unique diagnostic material are its availability and the noninvasive method of collection [13, 14]. Collection is fast, inexpensive, and safe. In addition, saliva as a “body mirror” can reflect the physiological and pathological state of the oral cavity. Therefore, it serves as a diagnostic and monitoring tool in many fields of science, such as medicine, dentistry, and pharmacotherapy [15]. By analyzing the image of saliva, we not only can assess the degree of caries risk but also obtain additional possibilities to diagnose specific diseases that give early symptoms in the mouth. In 2002, the National Institute of Dental and Craniofacial Research (NIDCR) eliminated all the obstacles by approving body fluids as a diagnostic tool to assess the state of health and disease [16].

## 2. Aim of the Study

The aim of this review is to emphasize the diagnostic value of saliva as a research material in the configuration of its structure and secretion disorders. This review summarizes the comprehensive literature search, which was conducted using databases, and presents the selected studies of the physical and chemical properties of saliva in the field of dental treatment in various physiological and pathological areas of human health.

## 3. Materials and Methods

**Figure 1:**
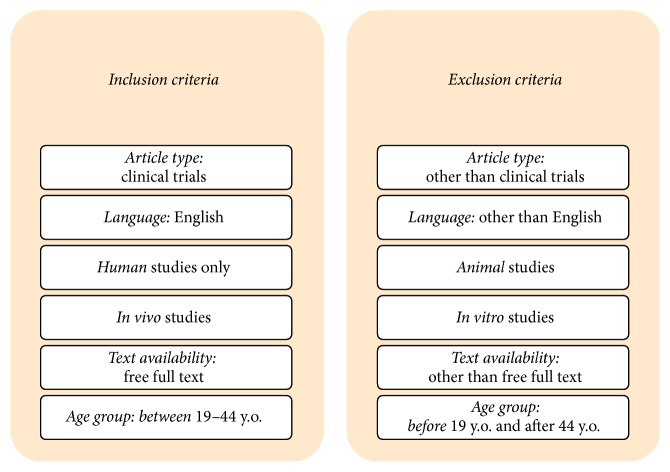
Criteria for including and excluding the correctness of qualifying the analyzed articles.

The selected literature covers the years 2000–2018. The data were obtained using the MEDLINE (PubMed) search engine, as well as an additional manual search. The results were evaluated and checked for the correctness of qualifying in accordance with inclusion and exclusion criteria (Figure 1). The analysis covered 77 articles selected from a group of 1986 publications and initially qualified for devising.

## 4. The Essence of the Matter

The analysis of blood, which is the most common preparation in clinical chemistry, aims to identify diseases and monitor the progress of treatment. However, medical personnel increasingly appreciate and use saliva as a diagnostic material. Saliva is a clinically informative biological fluid that can be used in innovative laboratory and clinical diagnostics, as well as in monitoring and treating patients with both oral and systemic diseases. The diagnostic use of saliva has attracted the attention of many researchers due to its noninvasive nature and relative simplicity of collection [17, 18].

Saliva as a diagnostic material is easy to collect, transport, and store. However, it should be remembered that samples of this material ought always to be collected from patients at the same time, preferably between 9:00 and 11:00 am (due to the highest physicochemical stability). A patient should not eat at least 90 minutes before the collection and stop taking medicines that affect salivary secretion 1 day before the procedure. Before sampling, the mouth should be rinsed with deionised water, and saliva should be collected for 10 minutes [19]. There are various methods of depositing saliva, depending on the type of material we want to obtain: the free flow of nonstimulated saliva from the mouth, spitting out using the Navazesh method [20] with little muscle stimulation, or sucking the saliva from the bottom of the oral cavity and its absorption [21]. In order to obtain stimulated saliva, we recommend chewing paraffin blocks or unflavoured and sugar-free chewing gum, which affects the secretion of saliva. The bacterial material and cellular impurities must be removed immediately after collecting the sample. The use of cotton filters for this purpose is not advisable, since hormones and proteins have an affinity for this type of material, and they may give false results or lower the diagnostic value of the test [22]. In order to determine the ionic composition and rheological properties of saliva, tests are carried out immediately after collecting the sample. In order to store saliva samples as a diagnostic material, they should be frozen, preferably in liquid nitrogen. Samples also ought to be collected into cooled containers. If samples are to be stored for a long time, the preferred temperature is −80°C. Potentially detrimental effects of freezing and defrosting can be compensated for by freezing samples at a temperature of −80°C, diluted in a ratio of 1 : 1 in glycerol [23].

Depending on the purpose of the material, glass or plastic containers are used, into which saliva can be drained, spat, or sucked. There are also absorption methods using commercially available kits, for example, Salivette. They include an absorbent cotton or synthetic cartridge, which the patient chews for a given period of time [24]. Because saliva sampling is noninvasive and gives an opportunity to carry out the procedure under various circumstances (large-scale studies, field tests, and the possibility of taking samples by people without medical training), it exceeds blood and urine tests in terms of usefulness. The simple, noninvasive method of obtaining the material for research and the repeatability of results make it useful to be applied in point-of-care tests [25].

**Figure 2:**
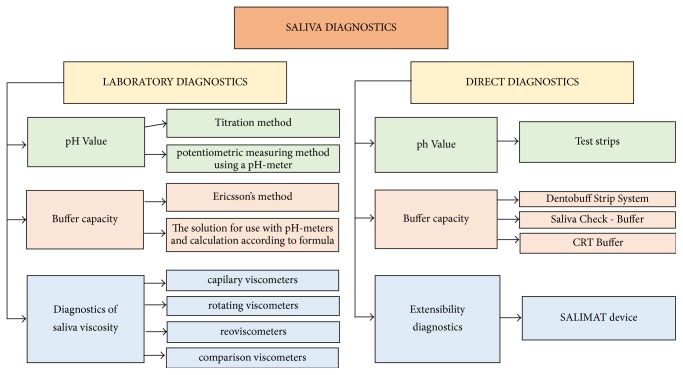
The original diagram of saliva diagnosis, which is also possible to be performed in the dental office.

Saliva diagnosis can be performed in the laboratory or directly in the dental office. Not only does the examination of the properties of this systemic secretion include the contained substances and chemical parameters, but also physical properties are increasingly explored (Figure 2).

### 4.1. Salivation Secretion Disorders

An adult person produces from 0.5 to 2 litres of saliva per day, but only 2–10% of the fluid is produced at night. During sleep, the rate of salivary secretion decreases to less than 0.25 ml/minute, while during chewing or speech these values oscillate around 10 ml/minute [26]. The basic salivary secretion is 0.33–0.55 ml/min on average and varies significantly between individuals, even under standard conditions. After strong excitatory stimulation, for example, under the influence of a food stimulus, the salivation can increase up to 1.5–2.3 ml/min, and after the administration of pharmacological agents, such as pilocarpine or methacholine, it reaches the value of 5.0 ml/min. The daily volume of saliva depends on the amount of sleep, the frequency and type of meals, and the action of emotional stimuli [27].

In their studies, Fallahi et al. proved the insufficiency of cohesion forces in situations where the lowest value of adhesion forces was caused by the reduction of salivation, showing that not only the quality but also the amount of saliva secreted in adults using mobile prosthetic restorations is important [28]. The discussed issues were also raised by Turner et al. [29].

In their studies conducted in patients with xerostomia, Davis et al. demonstrated an increase in salivary secretion after the use of a toothpick by 440% compared to the baseline, while the growth was by 628% in subjects using toothpicks containing additional spilanthol, which is a fatty acid amide of plant origin obtained from the electric daisy (*Acmella oleracea*) [30].

The analysis of saliva performed by Khanum et al. immediately before and after extracorporeal dialysis in patients with renal insufficiency and significant changes in the oral environment showed an immediate effect of the stimulation of salivary secretion after the procedure [31], which was also investigated by other scientists [32, 33].

Various studies using physical stimuli were conducted in order to help people suffering from xerostomia. Aparna et al. observed that the production of saliva by the parotid glands increased in 76% of patients suffering from xerostomia after the use of percutaneous electrical stimulation (TENS currents) [34, 35].

Xerostomia is also associated with the consumption of some drugs, for example, anticoagulants, antidepressants, antiretroviral, and nonsteroidal anti-inflammatory drugs, as evidenced by numerous reports [36]. López-Pintor et al. have shown that the condition is also very common in patients suffering from diabetes in whom a decreased flow of saliva may additionally occur [37–39]. The studies on the causes and risk factors of xerostomia conducted by Niklander et al. demonstrated that the disease was more frequent in women above 60 years of age and in people who took medications for a long time, while menopause was considered as one of the risk factors [40].

Hypersalivation is extremely troublesome for patients struggling with Parkinson's disease. Nowadays, three mechanisms leading to increased salivation have been identified in this disease: elevated salivary gland production, disturbances of salivary absorption in the bottom of the oral cavity, and disorders of oral saliva removal. In refractory schizophrenia, hypersalivation is most often caused by clozapine [41].

Botulinum toxin is one of the most effective treatments for increased saliva secretion. In their studies, Mazlan et al. attempted to determine the most effective dose of this neurotoxin in reducing salivation in adult Asians suffering from neurological diseases. A prospective, randomized, double-blind controlled trial was conducted, which lasted for 24 weeks. The reduction in secretion was greater in the groups that received higher doses. The group taking 200 units showed the greatest decrease in salivation before week 24 and reported the most significant improvement [42–45]. In children, however, botulinum and Kinesio Taping [46, 47] can be applied to reduce salivation.

### 4.2. Saliva pH

Laboratory tests focus not only on the determination of substances in saliva but also on the analysis of its parameters. The pH value is one of them.

Saliva is a slightly acidic (pH 6-7) secretory fluid whose main ingredient is water (99%). Saliva collected without stimulants, such as food, is hypotonic, and after stimulation, it becomes isotonic compared to plasma [48]. Saliva density is in the range of 1.002–1.012 g/ml, and its pH to a large extent depends on the rate of production. At night, when the salivation process is slower than that during the day, the pH reaches about 6.2–6.5. Saliva pH can increase up to around 8.0 due to the increased content of bicarbonate ions [49]. The concentration of hydrogen ions plays a significant role in the biophysicochemical processes taking place in the oral cavity. Saliva pH is not a constant value but undergoes significant changes under the influence of various factors (salivary secretion rate, daily cycle, diet, systemic diseases, and vegetative nervous system). The pH of mixed saliva is 6.38 (from 5.8 to 7.5) on average. The pH of whole saliva was shown to be higher in the morning than at midday and significantly higher after meals [48].

There was no relationship between the pH value and the age of patients; however, pH values differed in various areas of the oral cavity [50]. The highest value is found on the mucous membrane of the cheeks at the mouth of Stenson's duct, while the lowest value is found on the gums. There is the commonly known concept of critical pH, explaining the dissolution of enamel apatites at low concentration of hydrogen ions [51]. Maintaining the mineral balance between hydroxyapatite and saliva is important for the condition of the enamel. At pH = 5.5, the saliva is a saturated solution of calcium and phosphate ions, and under these conditions, these ions migrate to hydroxyapatites. When the pH drops, saliva becomes an unsaturated solution of these ions, which causes them to move in the opposite direction [5]. This leads to demineralization of the enamel, which stays unchanged for as long as these two processes remain in equilibrium. A drop in pH below the critical value (pH < 5.5) leads to superficial demineralization of the dental tissues [52]. Chemical dissolution of enamel hydroxyapatites without the use of bacteria is called enamel erosion. The erosion can be caused by acids supplied with food or internal acids formed in the body of patients suffering from bulimia, anorexia, and gastroesophageal reflux. The presence and intensity of erosive changes depend on the pH of saliva, its secretory capacity, buffering, composition, and the quality and duration of the action of a damaging factor [53].

The use of various oral care products also has an influence on pH. A phase IV clinical trial with three age groups was designed in order to analyze the effect of two rinses on saliva pH and to correlate the outcomes with age, buffer capacity, and salivary flow rate in healthy volunteers. A sudden significant increase in saliva pH was observed immediately after rinsing, while after 15 minutes the value dropped to an almost stable level. A huge increase in saliva pH value after using commercial rinses proves that saliva is a dynamic system and that the body is able to react to the stimulus by changing the content of this fluid. The results of this study increase the importance of in vivo measurements and strengthen the concept of the protective action of saliva [54].

The analysis of the latest literature reports led to the conclusion that the potentiometric method using pH meters is the most common laboratory technique for measuring saliva pH and that this parameter should be measured immediately after collecting the material [55]. There are also possibilities of direct diagnostics, for example, in the dental chair. This is a very easy and fast method of saliva pH diagnostics. Special test strips are used for this purpose; pH ought to be measured immediately after the material's collection; the result is obtained 10 seconds after the application of the material to the test strip, and data are read from the colour scale to an accuracy of 0.2.

### 4.3. Saliva Buffer Capacity

Given the modern methods of testing secretions, such as saliva, we should mention the possibilities of measuring buffering capacity. Hydrogencarbonate ions (HCO3−) are the most important in maintaining the homeostasis, chemical stability, and buffer capacity of saliva. Their concentration increases with the volume of saliva, proportionally reaching a peak of 40–60 mmol/l, which clearly exceeds the concentration of this anion in the blood plasma [56]. An increase in the HCO3− concentration is combined with a growth of salivary pH. This value is about 5.6 at the basal secretion and up to 7.8 during maximal secretion. In unstimulated saliva, the concentration of H+ ions is relatively constant. The stable pH level is maintained also thanks to buffering agents like bicarbonates, phosphates, proteins, free amino acids, ammonia, and urea [57].

The main buffering systems are bicarbonates, phosphates, and proteins. Bicarbonates are secreted only by large salivary glands, and their concentration depends on the rate of secretion. The second buffering agent is phosphate, whose concentration decreases with the increasing secretion rate. Because, contrary to the level of phosphates, the concentration of bicarbonates grows with the rate of saliva secretion, they are responsible for about 50% of the resting saliva buffer capacity and about 80% of stimulated capacity. Proteins and free amino acids have little effect on the buffering capacity of saliva [26].

In their studies, Nishihara et al. evaluated the effect of lactic acid bacteria* Lactobacillus salivarius* on the risk factors of caries. The participants were randomly divided into four groups and took* L. salivarius* WB21,* L. salivarius* TI 2711, Ovalgen® DC, or xylitol-containing tablets. They placed the pill on the tongue for a few minutes and allowed it to dissolve. The levels of streptococci and lactobacilli as well as the salivary flow, pH, and buffering capacity were evaluated before and after taking appropriate tablets. The level of streptococci was evaluated using Dentocult® SM Strip mutans, and the level of lactobacilli waas evaluated with Dentocult LB; the pH values were investigated using the CheckBuf test kit and the amount of salivary flow was assessed by a gum test. No significant differences were found between the groups in terms of the salivary flow and pH. The saliva buffering capacity increased significantly in the group of* L. salivarius* and Ovalgen® DC compared to the xylitol group [58].

In his study, Mummolo et al. assessed the bacterial levels of* Streptococcus mutans* and* Lactobacillus* spp., dental plaque, salivary flow, and buffer capacity of saliva before and during orthodontic treatment. The plaque index (PI) increased over time in each group, just like the saliva flow, and mainly in subjects treated with self-ligating brackets, suggesting a difference between these devices and conventional systems. The authors of the study call for periodic microbiological monitoring and the control of salivary parameters during orthodontic treatment [59].

Diagnosis of saliva buffer capacity can be carried out in the laboratory or, as it more often happens, in a dental office through direct diagnosis using special test kits. The saliva buffer capacity can be tested in three ways:

1. The Ericsson method: it is certainly the most accurate of the three methods mentioned; however, it is also the most time-consuming. A saliva sample is collected from the patient and then transferred and processed in a medical laboratory. The results are then sent back to the dental practitioner for consideration. Precisely, 1 ml of freshly collected saliva should be transferred to 3 ml of HCl (0.005 M for stimulated saliva and 0.0033 M for resting saliva). To prevent the foaming of the solution, add one drop of 2-octanol and mix it for 20 minutes to remove CO_2_. The final pH of the solution is then measured using a pH meter
2. The Dentobuff Strip System method: this method is much faster; a result can be obtained within a 5-minute period; however, this is neither as accurate nor as comprehensive as the aforementioned Ericsson method. Buffer capacity is determined based on the colour of the test strip 5 minutes after the application of a saliva drop onto the strip
3. The Saliva Check-Buffer method: it is decidedly the fastest of the three methods with results being available after only two minutes. Interestingly, it is also more precise than the Dentobuff Strip System due to it having more test areas. Buffering capacity is determined based on the colour of the test strip windows two minutes after the application of saliva is applied to the strip.

## 5. Elasticity of Saliva

Mucins are mainly responsible for the elasticity of saliva. They are compounds rich in proline, glycoproteins, and electrolytes, as well as water, and are considered protective agents of the oral mucosa. These substances are one of the most important proteins in the mouth. In nonstimulated saliva, they constitute 20–30% of the total amount of proteins. With their increasing amount, the density and viscosity of the secretion grow. The viscosity of saliva depends primarily on the MG1 glycoprotein [60, 61].

No method has been introduced into general use which would allow for the unambiguous qualitative and quantitative diagnosis of saliva in terms of the biophysical properties mentioned above. From a technical point of view, a determination of the viscosity of saliva in a laboratory test does not cause major problems because viscosimeters are used for this evaluation. Different models of the tools are distinguished, capillary, rotational, with a falling ball, and comparative, allowing for the measurement of relative viscosity. Another approach to assess the biomechanical properties of saliva is to measure its elasticity [62].

It is known that carious lesions in the teeth can in various ways affect the properties of saliva. Aminabadi et al. examined how the elimination of active tooth decays in people with more than five tooth caries surfaces influenced saliva parameters. The selected parameters were evaluated in the samples of unstimulated saliva. One month after the treatment of caries, during which all the affected surfaces were removed, saliva samples were collected and reanalyzed. The salivary viscosity significantly decreased, while the buffering capacity and saliva pH significantly increased. It should be concluded, therefore, that tooth caries can be prevented by improving the quality of saliva, including its physical parameters [63–65].

**Figure 3:**
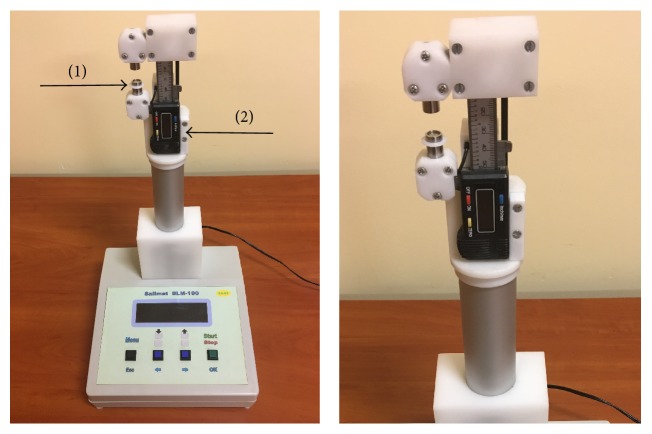
SALIMAT-SLM-100 (Poland), a prototype device for measuring the direct viscosity of saliva. (1) A saliva container. (2) Electronic measurement display.

SALIMAT (Poland), which is a prototype device used to test the direct viscosity of saliva, consists of a digital calliper as a supporting structure and reader to examine saliva (Figure 3). This test is a simple and effective measurement method and generally comes down to the determination of the length of the stretched saliva sample, of a specified volume, until it is broken. The device allows for the manual and automatic measurement of the length at which the sample breaks. It can be synchronized with a computer or work independently. The value at which the length of the saliva sample is broken can be read on the device display with an accuracy of ±0.001 mm. The moving part of the device is equipped with a drive wheel that allows it to move smoothly, which is important for automatic and precise measurement. Special overlays are mounted coaxially on the arms of the calliper. The upper one is formed by a flat-ended cylinder with a diameter of 9 mm, and in the lower one having a diameter of 10 mm a recess is milled (diameter 8 mm, depth 1 mm, and volume 50.26 mm^3^), and here the sample of saliva is placed.

## 6. Determination of Hormones

### 6.1. Cortisol

The determinations performed in saliva can be used to diagnose infectious diseases, autoaggressive diseases, and cancer, as well as endocrine and cardiac disorders. Saliva can also be applied as a material in the examination of drug levels or tests for the presence of narcotics. Cortisol is a hormone most commonly determined in saliva. The diagnosis and monitoring of the therapy are based on changes in the levels of concentrations of this hormone [66]. Cortisol, which is the most important glucocorticoid, is synthesized by the adrenal cortex. Its production is regulated by the ACTH hormone, secreted from the pituitary gland, which in turn is controlled by CRH released from the hypothalamus, ACTH-releasing hormone. Over 90% of serum cortisol is connected with corticosteroid binding globulin (CBG, transcortin). During pregnancy or oestrogen therapy, the CBG concentration decreases, and consequently the total cortisol concentration increases. Free cortisol is biologically active; it affects the metabolism of proteins, fats, and carbohydrates and has anti-inflammatory and antiallergic properties. Cortisol has an impact on carbohydrate, fat, protein, calcium, and phosphate metabolism [67].

Both increased and decreased levels of cortisol have a negative impact on the body and produce various symptoms and consequences. Cortisol tests are closely related to oral health. Lopez-Jornet et al. demonstrated that patients with lichen planus located in the oral cavity suffered from sleep disturbances and had higher levels of cortisol and proteins compared to the control group of healthy patients [68].

Cortisol determination is recommended if we suspect the excessive production or the lack of cortisol production by the adrenal cortex (in Cushing's and Addison's disease, hypothyroidism, hypoplasia of the adrenal glands, or neoplasms). The secretion of cortisol shows large daily changes; the highest values are observed early in the morning and the lowest ones around midnight. Thus, a single measurement of cortisol is of little importance—we should take samples in the morning and in the evening. Cortisol determination also plays an important role in certain stimulatory and suppressive tests that are used to examine the activity of the hypothalamic-pituitary-adrenal cortex axis [69].

In order to test saliva, we should collect the fluid in special tubes and centrifuge it. The examination is performed in 7 saliva samples collected throughout the day at specific times. Seven determinations throughout the day allow for the accurate assessment of changes in cortisol levels during the day. The measurement of cortisol in saliva is a simple, convenient, and accurate technique with a potential value in monitoring patients with hypercortisolemia. This analysis shows the late-night changes in salivary cortisol. Cortisol, determined in salivary samples, can be a simple, convenient biomarker for diagnosing and assessing the response to treatment in patients with Cushing's disease [70, 71]. Elias et al. also proved that salivary cortisol was more sensitive and reliable than urine cortisol, and it should be the basic biochemical test in diagnosing this disease [72].

Lokhmatkina et al. and Trickett et al. focused on the measurement of cortisol in salivary samples obtained from women who were victims of domestic violence and sexual abuse. The authors claim that this measurement may allow for a better understanding of the pathophysiological mechanisms of mental disorders related to violence in women and inform researchers and practitioners about the possibility of using salivary cortisol as a biological marker of prognosis, diagnosis, and treatment in those who are victims of this abuse [73, 74].

In their research, Peterson et al. confirmed the fact that the analysis of cortisol level can be used in psychiatric and psychological examinations. In the laboratory protocol, men were exposed to neutral stimuli causing negative and positive effects. The concentration of cortisol in saliva was measured during the whole laboratory procedure. The results of this study were consistent with the view that male sexual aggression is connected with physiological hyporeactivity and physiological profile associated with psychopathic behaviours and features [75]. Feinberg et al. confirmed these reports by analyzing the level of cortisol in the saliva of couples expecting their first child and who were experiencing emotional violence in the relationship. In these studies, which focused on the assessment of the hypothalamic-pituitary-adrenal axis activity, the authors showed that a persistent increase in the activity of this structure in men, with the simultaneous reduction of this activity in women, led to the onset of male violence in a later period [69].

Vreeburg et al. analyzed the level of cortisol in patients with various anxiety disorders. Three groups of patients were compared: control, without psychiatric disorders, and remitted, with no current anxiety disorder and with current anxiety disorder. Cortisol levels were measured in seven saliva samples. A slight but extremely significant difference was demonstrated in patients suffering from panic attacks with coexistent agoraphobia and depression [76].

The level of cortisol also changes under the influence of various stressful situations and the preparation for such events. This was confirmed by Meunier et al. in a study that concerned, among others, the analysis of salivary cortisol levels before, during, and after a simulated task related to intense emotional arousal. Communication skills training has an effect on physiological arousal. After simulated training, the cortisol level significantly increased compared to the control group [77]. Bedini et al. subjected a group of emergency response coordination centre operators to similar examinations. They noted higher levels of cortisol during shift work and in the subgroup of men. Higher cortisol levels are also observed during a serious life-threatening condition [78].

Tecles et al. and Preuß et al. used biomarkers contained in saliva to determine the level of students' stress related to public speaking, as well as a written exam. They showed that the highest concentration of cortisol was observed 20 minutes after the end of the presentation, and it had no connection with the level of stress indicated by students in the surveys, gender, or the quality of the presentation. In the case of written exams, the level of cortisol was high already on the day before the exam and dropped immediately after its completion [79, 80].

By means of serial cortisol measurements, we can also determine the adrenal cortex response to CRF [81]. Huhn et al. examined the level of this hormone in women during labour. They measured the level of cortisol in saliva before and after the occurrence of an acute painful stimulus. This is the measurement of the so-called biological response to pain. The response to pain was generally lower than expected and completely resolved within 72 hours [82].

The examination of salivary cortisol is also important in research into numerous disorders involving hygiene and quality of life. On the other hand, Lipschitz et al. proved that educational training on sleep hygiene caused a decrease in the cortisol levels in salivary samples in patients after cancer [83].

### 6.2. Leptin

Leptin is a substance that has been frequently tested and measured over the past few years. It is a protein secreted mainly by fat cells, and as a hormone it plays a role in the regulation of food intake and energy management. Gröschl et al. showed that leptin is also produced, stored, and secreted by the salivary glands, and its level increases with the flow of saliva [84].

Similar conclusions were presented by Jayachandran et al. who proved that obese people had higher levels of leptin in saliva. Their research also demonstrated a close relationship between the level of leptin and the range of movement of the teeth supplied with orthodontic devices. Smaller dislocations were reported in obese subjects than in those with normal Body Mass Index (BMI) [85].

Schapher et al. also demonstrated a higher level of leptin in all types of salivary gland neoplasms and thus indicated that it was a marker in the diagnosis of salivary gland tumours [86]. However, lower levels of the hormone were observed in patients with advanced periodontal disease [87].

The studies conducted by Rodrigues et al. on the level of leptin in saliva and taste sensation demonstrated that this level was higher in a group of girls but only those with normal body weight and a reduced sense of sweet taste. In a group of boys who were more sensitive to the bitter taste, elevated levels of this hormone were reported. The authors also found a tendency to higher concentrations of this hormone in obese people [88].

## 7. Determination of Enzymes

### 7.1. Amylase

Amylase is another substance very often determined in saliva in the laboratory. It is a protein that in human saliva is responsible for the formation of the glycoprotein complex within the pellicle which is formed on the surface of the teeth immediately after cleaning. It has a high affinity for bacteria living in the mouth and is associated with them by adhering to the surfaces in the mouth [89]. It also takes part in the initial digestion of food and helps remove carbohydrate fibres from the interdental spaces. Contreras-Aguilar et al. revealed that both activity and concentration of amylase vary depending on the stress situation and the method of determination and interpretation. These differences may result from many factors, such as changes in the salivary flow, the concentration of all salivary proteins, and various stressful situations [90]. Stress and pain are often interrelated events. Investigators have attempted to distinguish them using a variety of model systems that induce either stress or pain and subjects are monitored for changes in salivary biomarkers. A typical marker that has been identified is salivary amylase.

In their studies focused on the diagnosis of saliva in physically active people, Rutherfurd-Markwick et al. showed that the resting activity of amylase was higher in a group of women than in men. However, in men, a relationship was observed between the secretion of amylase and cortisol [91].

Among members of a Paralympic swimming team, Edmonds et al. reported an increase in the level of amylase during 14 weeks of preparation for international competitions, probably due to the impact of the parasympathetic system [92].

Higher levels of mothers' amylase secretion after caesarean section and with direct intraoperative contact with the child were demonstrated, compared to a group of mothers who had contact with their babies only after the procedure [93].

It was also shown that the consumption of coffee, albeit not being a stress factor, resulted in an amylase activity increase in saliva, without changes in the level of cortisol, which may suggest the activation of the sympathetic nervous system. As the level of amylase increases after drinking coffee without a simultaneous rise in cortisol, the authors of the abovementioned publication suspect that it may have antistress properties [94].

Not only caffeine but also methamphetamine (METH) aroused the interest of researchers in terms of the influence on salivary amylase. Haile et al. assumed that METH must affect the concentration of amylase because it activates the sympathetic system by increasing the central and peripheral concentration of norepinephrine. It should be noted that it is a biomarker of the sympathetic system activation, which correlates with the level of norepinephrine in plasma. The researchers managed to show that METH increased the level of amylase and was dependent on age, BMI, and the amount of METH taken per kilogram of body weight. Thus, they demonstrated that the activity of amylase, which is a peripheral norepinephrine biomarker, is closely related to the subjective effects of METH [95].

The results of amylase determination also revealed changes in the profile of salivary proteins in morbidly obese women who were qualified for bariatric surgery. Lower levels of amylase were noted in a group of obese women who underwent surgery; the activity of this protein was also lower than that in a group of obese patients [96].

### 7.2. Lysozyme

Lysozyme is an enzyme originating from the large salivary glands, gingival fluid, and digested leukocytes. Lysozyme is also known as muramidase or N-acetylmuramide glycanohydrolase, which is an antimicrobial enzyme produced by animals which forms part of the innate immune system. Lysozyme is a glycoside hydrolase that catalyses the hydrolysis of 1,4-beta-linkages between N-acetylmuramic acid and N-acetyl-D-glucosamine residues in peptidoglycan, which is the major component of Gram-positive bacterial cell walls. This hydrolysis in turn compromises the integrity of bacterial cell walls causing lysis of the bacteria. In humans, the lysozyme enzyme is encoded by the LYZ gene. Elevated salivary lysozyme levels, a biomarker for oral infection and hyperglycemia, have also shown a significant association with hypertension, an early stage of cardiovascular disease.

Gillum et al. also demonstrated that overproduction of leukocytes in saliva is not necessary to increase the amount of lysozyme [97]. Kim et al. determined the effect of xylitol and sorbitol on lysozyme activity. Both analyzed carbohydrates inhibited the enzymatic activity of lysozyme in a solution of whole saliva, while they did not affect the properties of this protein on the surface of enamel hydroxyapatites [98].

In research on new drugs aimed at inhibiting the growth of cariogenic colonies of bacteria living in the oral cavity, Tonguc-Altin et al. proved that, thanks to the appropriate carriers and in combination with lactoferrin, lysozyme can be used to reduce the amount of* S. mutans* and* L. acidophilus* [99].

Studies showed that, in diabetes, which is caused by increased glucose (Glc) concentration, glycation of proteins, including lysozyme, leads to the formation of advanced glycation end products (AGEs), which in turn significantly increases the risk and pace of complications development. Mirmiranpour et al. demonstrated in their work the possibility of inhibiting glycation of lysozyme by L-lysine, which gives an opportunity to alleviate pathological changes in this disease [100].

In a study on salivary defence proteins in women with or without human papilloma virus (HPV) infection, Haukioja et al. proved that the level of this protein was higher in patients with the chronic infection and that the HPV infection of the oral cavity might be associated with the elevated concentration of IgG and lysozyme [101].

In their research, Ligtenberg et al. have demonstrated that physical effort also affects the flow of saliva and the secretion of proteins (including lysozyme), amylase, and 5B mucin. Exercises of moderate intensity increase the levels of proteins and lysozyme, as well as amylase and 5B mucin. The flow of saliva also improves. On the other hand, very strenuous effort leads to a reduction in the amount of proteins in the secretion [102].

## 8. Determination of Proteins

### 8.1. Chromogranin A

Chromogranin A is an acidic secretory protein that can be found in many types of neuroendocrine tissues. It is secreted by sympathetic nerves together with catecholamines and can be used as a marker for sympathetic nervous system activity [103, 104]. Chromogranin A (CgA) is produced in submandibular salivary glands; therefore, taking the sample of material to determine its level is simple, fast, and painless for the patient [105]. A circadian rhythm can be observed in the secretion of this protein. The highest values were recorded around 11:00 pm and the lowest ones at about 8:00 am [106].

In their research, Yoto et al. investigated the antistress effectiveness of two kinds of green tea against a mental stress task load. A group of patients participating in the study drank warm water, green tea (Sagara), and shaded white tea. Saliva was collected before the fluid intake and after mental stress load tasks. Their studies showed that concentration of CgA in saliva increased after mental stress, but drinking green tea can inhibit the growth [107].

In their studies, Matsumoto et al. examined the soothing effects of fragrance from yuzu. The salivary CgA was used as a stress marker showing the activity of a sympathetic nervous system. The study group was exclusively women. Participants were examined twice, firstly using the yuzu scent and secondly using unscented water as a control in the follicular phase. They proved that inhalations of the yuzu scent significantly decreased salivary CgA levels [108].

### 8.2. Immunoglobulin A

Immunoglobulins A are an immunoglobulin class that is characterized by the presence of a heavy *α* chain in its structure. The main physiological role of IgA is to participate in immune reactions within the mucous membranes. The amount of IgA synthesized in the human body throughout the day is greater than all other immunoglobulins combined. Although the concentration of this immunoglobulin in the blood plasma is small, the vast majority of it is secreted on the surface of mucous and serous membranes. There are two IgA subclasses, IgA1 and IgA2, which can be produced as monomeric or dimeric form. The secretory IgA (sIgA) is dimeric form of this immunoglobulin. sIgA can be found in mucous secretions, like saliva, tears, sweat, and so forth [109]. It is produced by B-lymphocytes adjected to the mucosal cells, transported through cells, and released into the secretions. The highest levels of sIgA in humans can be found in the minor salivary glands [110]. The secretory circadian rhythm reaches the highest level in the morning and the lowest level in the evening [111]. Stress affects the secretion of sIgA in saliva. The level of this immunoglobulin also depends on the saliva flow rates [112].

Research carried out by van Anders has shown that chewing gum significantly decreased saliva production time by 3–6 min, but it also leads to lowering the IgA level, in both men and women groups [113].

Laurent et al. in their studies focused on understanding the effect of stress factors on secretion of sIgA in children and adolescents. In saliva samples obtained after exposure to a series of performance or interpersonal stressors in laboratory sessions, the levels of sIgA, cortisol, and alpha amylase were determined. Participants displayed SIgA reactivity to and recovery from acute stress. The relations of sIgA with cortisol and alpha amylase reflected different forms of cross-system linkage. Youth SIgA trajectories followed a normative pattern of reactivity and recovery around the stressors; however, these responses were blunted in young people with higher externalizing scores. In contrast to SIgA, neither cortisol nor sAA related significantly to behavioral problems [114].

## 9. Conclusions

Even though saliva is not a popular body fluid, more and more dentists, internists, paediatricians, pharmacologists, clinical and forensic pathologists, endocrinologists, immunologists, psychologists, and dentists are discovering that the liquid is an easily available, noninvasive diagnostic tool. Newly emerging and fast-growing technologies, such as the latest point systems, RNA sequencing, and fluid biopsy, can provide new diagnostic solutions in the field of saliva diagnostics. These recent advances have broadly widened the possibilities of saliva testing in the oral cavity, making clinical salivary diagnosis a reality that can be very precise and useful in the assessment of oral health. In addition, it should be noted that the determination of chemical and physical saliva parameters can be effectively performed in the patient's presence in the dental office.
